# Use of CPAP as an alternative to the apnea test during the determination of brain death in hypoxemic patients. Report of two cases

**DOI:** 10.5935/0103-507X.20200032

**Published:** 2020

**Authors:** Glauco Adrieno Westphal, Veviani Fernandes, Verônica Westphal, Jessica Cangussu Fonseca, Luciano Rodrigues da Silva, Jorge Luis dos Santos Valiatti

**Affiliations:** 1 Unidade de Terapia Intensiva, Hospital Municipal São José - Joinville (SC), Brasil.; 2 Unidade de Terapia Intensiva, Centro Hospitalar Unimed - Joinville (SC), Brasil.; 3 Unidade de Terapia Intensiva, Hospital Padre Albino - Catanduva (SP), Brasil.; 4 Unidade de Terapia Intensiva Pediátrica, Centro Hospitalar Unimed - Joinville (SC), Brasil.; 5 Medicina Intensiva, Faculdades Integradas Padre Albino - Catanduva (SP), Brasil.

**Keywords:** Apnea, Sleep apnea syndromes, Brain death, Respiratory insufficiency, Continuous positive airway pressure, Hypoxia, Electrical impedance, Tomography/methods, Apnea testing, Apneia, Síndromes da apneia do sono, Morte encefálica, Insuficiência respiratória, Pressão positiva contínua nas vias aéreas, Hipóxia, Impedância elétrica, Tomografia/métodos, Teste de apneia

## Abstract

The apnea test, which involves disconnection from the mechanical ventilator, presents risks during the determination of brain death, especially in hypoxemic patients. We describe the performance of the apnea test without disconnection from the mechanical ventilator in two patients. The first case involved an 8-year-old boy admitted with severe hypoxemia due to pneumonia. He presented with cardiorespiratory arrest, followed by unresponsive coma due to hypoxic-ischemic encephalopathy. Two clinical exams revealed the absence of brainstem reflexes, and transcranial Doppler ultrasound revealed brain circulatory arrest. Three attempts were made to perform the apnea test, which were interrupted by hypoxemia; therefore, the apnea test was performed without disconnection from the mechanical ventilator, adjusting the continuous airway pressure to 10cmH2O and the inspired fraction of oxygen to 100%. The oxygen saturation was maintained at 100% for 10 minutes. Posttest blood gas analysis results were as follows: pH, 6.90; partial pressure of oxygen, 284.0mmHg; partial pressure of carbon dioxide, 94.0mmHg; and oxygen saturation, 100%. The second case involved a 43-year-old woman admitted with subarachnoid hemorrhage (Hunt-Hess V and Fisher IV). Two clinical exams revealed unresponsive coma and absence of all brainstem reflexes. Brain scintigraphy showed no radioisotope uptake into the brain parenchyma. The first attempt at the apnea test was stopped after 5 minutes due to hypothermia (34.9°C). After rewarming, the apnea test was repeated without disconnection from the mechanical ventilator, showing maintenance of the functional residual volume with electrical bioimpedance. Posttest blood gas analysis results were as follows: pH, 7.01; partial pressure of oxygen, 232.0mmHg; partial pressure of carbon dioxide, 66.9mmHg; and oxygen saturation, 99.0%. The apnea test without disconnection from the mechanical ventilator allowed the preservation of oxygenation in both cases. The use of continuous airway pressure during the apnea test seems to be a safe alternative in order to maintain alveolar recruitment and oxygenation during brain death determination.

## Abstract (pt)

O teste de apneia com desconexão do ventilador mecânico representa riscos durante a determinação da morte encefálica, especialmente em pacientes hipoxêmicos. Descrevemos a realização do teste de apneia sem desconexão do ventilador mecânico em dois pacientes. O primeiro caso é o de um menino de 8 anos, admitido com hipoxemia grave por pneumonia. Apresentou parada cardiorrespiratória, seguida de coma não responsivo por encefalopatia hipóxico-isquêmica. Dois exames clínicos constataram ausência de reflexos de tronco, e o Doppler transcraniano revelou parada circulatória encefálica. Realizaram-se três tentativas de teste de apneia, que foram interrompidas por hipoxemia, sendo então realizado teste de apneia sem desconexão do ventilador mecânico, ajustando a pressão contínua nas vias aéreas em 10cmH2O e fração inspirada de oxigênio em 100%. A saturação de oxigênio manteve-se em 100% por 10 minutos. A gasometria pós-teste foi a seguinte: pH de 6,90, pressão parcial de oxigênio em 284,0mmHg, pressão parcial de dióxido de carbono em 94,0mmHg e saturação de oxigênio em 100%. O segundo caso é de uma mulher de 43 anos, admitida com hemorragia subaracnóidea (Hunt-Hess V e Fisher IV). Dois exames clínicos constataram coma não responsivo e ausência de todos os reflexos de tronco. A cintilografia cerebral evidenciou ausência de captação de radioisótopos no parênquima cerebral. A primeira tentativa do teste de apneia foi interrompida após 5 minutos por hipotermia (34,9oC). Após reaquecimento, o teste de apneia foi repetido sem desconexão do ventilador mecânico, evidenciando-se manutenção do volume residual funcional com tomografia de bioimpedância elétrica. Gasometria pós-teste de apneia apresentava pH em 7,01, pressão parcial de oxigênio em 232,0mmHg, pressão parcial de dióxido de carbono 66,9mmHg e saturação de oxigênio em 99,0%. O teste de apneia sem desconexão do ventilador mecânico permitiu a preservação da oxigenação em ambos os casos. O uso de pressão contínua nas vias aéreas durante o teste de apneia parece ser uma alternativa segura para manter o recrutamento alveolar e a oxigenação durante determinação da morte encefálica.

## INTRODUCTION

The determination of brain death (BD) requires the findings of unresponsive coma, absence of brainstem reflexes, and apnea and complementary examination demonstrating electroencephalographic silence or absence of intracranial blood flow.^(1,2)^

The apnea test aims to determine the absence of respiratory movements through maximal stimulation of the bulbar respiratory center when partial pressure of carbon dioxide (PaCO_2_) levels greater than 55mmHg are obtained.^(1)^ Classically, the apnea test is performed by disconnecting the patient from the mechanical ventilator (MV) and instilling 6L/minute of oxygen with a catheter positioned at the height of the tracheal carina or 12L/minute via a T-piece connected to the orotracheal tube. However, disconnection from the MV can cause severe hypoxemia and hemodynamic instability.^(1-3)^

Even in patients with normal lungs, the apnea test with MV disconnection can lead to atelectasis and hypoxemia during and after the test. In addition, acute respiratory distress syndrome (ARDS) occurs frequently in neurocritical patients, and the *post hoc* analysis of a multicenter clinical trial showed that 26.2% (195/772) of patients with suspected BD had a partial pressure of arterial oxygen/fraction of inspired oxygen (PaO_2_/FiO_2_) < 200.^(4)^ In these patients, respiratory conditions may not allow safe disconnection from the MV for enough time to generate the necessary increase in PaCO_2_ to determine BD.^(3)^ In addition, the risk of absorption atelectasis caused by preoxygenation with 100% FiO_2_ must be considered, as well as the administration of pure oxygen during the test.^(5)^

The maintenance of alveolar recruitment during the apnea test with the application of continuous positive airway pressure (CPAP) may help prevent hypoxemia and is associated with higher PaO_2_/FiO_2_ values at the end of the test than those observed with the conventional method.^(6,7)^

Rare reports highlight the possibility of using CPAP as a safe alternative for the apnea test.^(8-12)^ Additionally, the use of CPAP during the apnea test in patients with normal lungs can assist in lung preservation strategies for donation purposes.^(13-15)^

To contribute to the safety of BD determination in hypoxemic patients, we describe two cases for which the apnea test by CPAP was performed as an alternative to maintain alveolar recruitment and avoid hypoxemia when MV disconnection was not safe.

## CASE REPORTS

### Case 1

**Figure 1:**
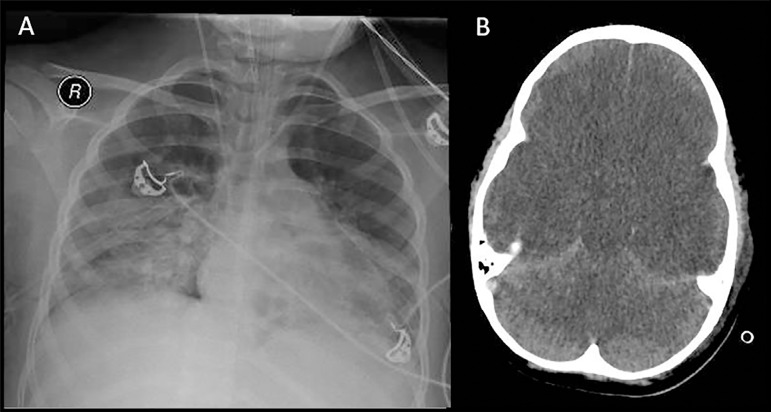
Imaging exams. (A) Chest radiograph showing diffuse pulmonary infiltrate. (B) Brain computed tomography showing cerebral edema.

An 8-year-old boy with ARDS and septic shock due to community-acquired pneumonia admitted to the intensive care unit (ICU) presented with cardiorespiratory arrest attributed to hypoxemia. Upon admission to the ICU, he had an arterial oxygen saturation (SaO_2_) of 95%, with an FiO_2_ of 100% and positive end-expiratory pressure (PEEP) of 10cmH_2_O, diffuse pulmonary infiltrate on chest radiography (Figure 1A) and blood pressure of 105/60mmHg with noradrenaline at 0.2mcg/kg/minute. The patient had bilateral fixed dilated pupils, and the corneal and cough reflexes were absent. Computed tomography of the brain revealed diffuse cerebral edema with signs of intracranial hypertension (Figure 1B). After 24 hours, unresponsive coma, absence of all brainstem reflexes in two clinical exams and absence of intracranial blood flow on transcranial Doppler ultrasound were observed. The apnea test remained to complete the BD determination.

The apnea test attempts proceeded as follows. Ten minutes after preoxygenation with an FiO_2_ of 100% and PEEP of 10cmH_2_O, an SaO_2_ of 100% was obtained. Blood pressure was 104/62mmHg, with noradrenaline at 0.16 mcg/kg/minute and vasopressin at 0.0003UI/kg/minute. The apnea test was started with disconnection from the MV and oxygen infusion at 6 L/minute at the tracheal carina level. The test was interrupted by a drop in SaO_2_ to less than 85%. Four new attempts were interrupted due to hypoxemia.

**Figure 2:**
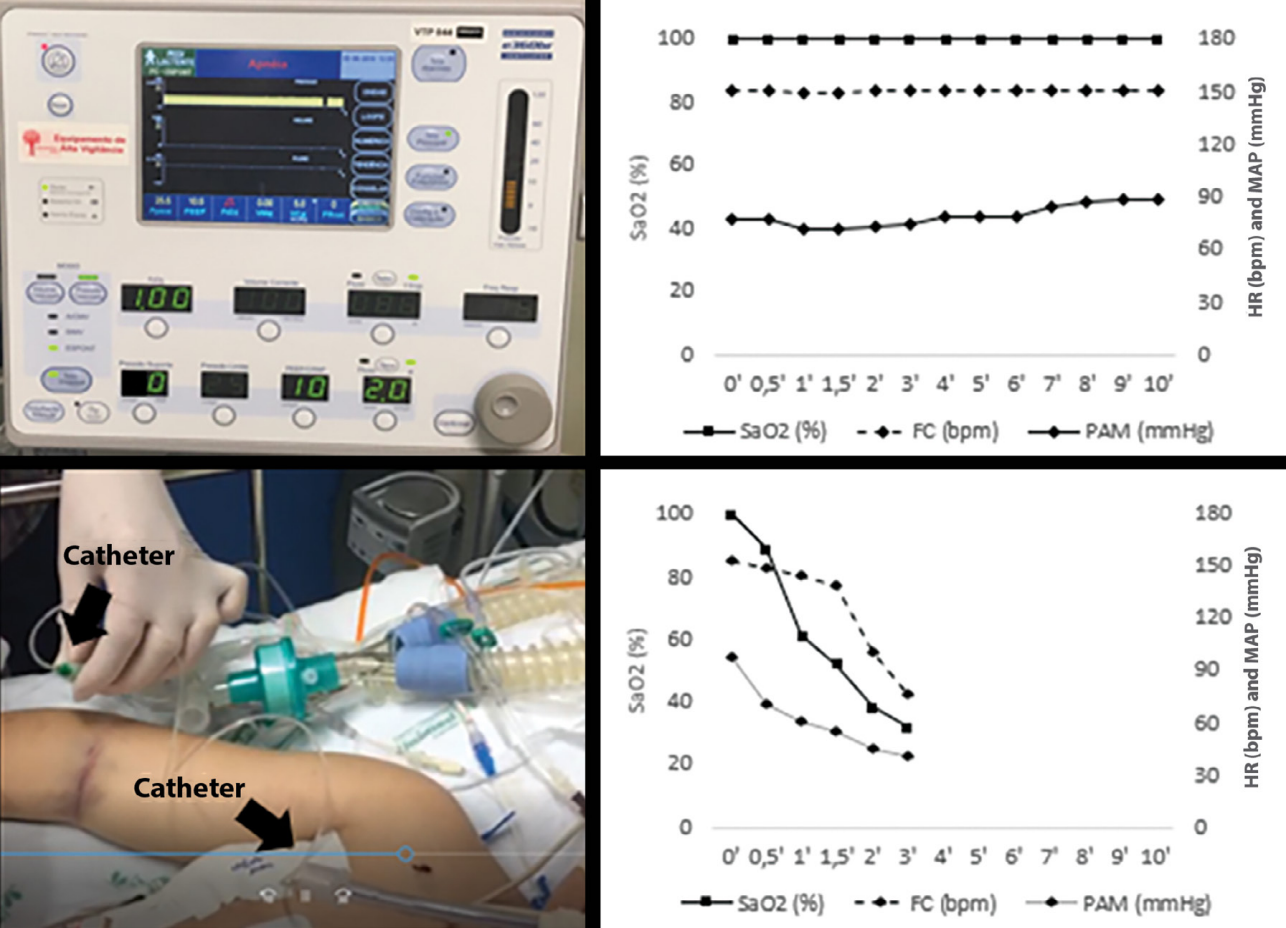
Heart rate, mean arterial pressure and arterial oxygen saturation pattern after disconnection from the mechanical ventilator (top) and oxygen infusion at 6 L/minute using a catheter at the tracheal carina level (bottom). SaO2 - arterial oxygen saturation; HR - heart rate; MAP - mean arterial pressure.

An apnea test without MV disconnection was performed after preoxygenation at 100% FiO_2_ for 10 minutes, at which time a new pretest blood gas analysis was obtained: pH, 7.15; PaO_2_, 254.0mmHg; PaCO_2_, 43.0 mmHg; bicarbonate (HCO_3_), 14.4mmol/L; and SaO_2_, 99.0%. The MV was set in spontaneous mode with the following adjustments: FiO_2_, 100%; CPAP, 10cmH_2_O (previous PEEP level); and reserve ventilation disabled. SaO_2_ was maintained at 100% during the 10 minutes of apnea. A mean arterial pressure (MAP) ≥ 65mmHg was maintained, with no significant change in heart rate (HR) (Figure 2). The result of the posttest blood gas analysis (pH, 6.90; PaO_2_, 284.0mmHg; PaCO_2_, 94.0mmHg; HCO_3_, 18.4mmol/L; and SaO_2_, 100%) and the absence of respiratory movements allowed determining patient death.’

There was no consent for organ donation. Artificial support therapy was withdrawn prior to delivery of the body to the family.

After disconnecting the MV, a catheter was inserted at the level of the tracheal carina with an oxygen flow of 6L/minute. Despite oxygen supplementation, there was a decrease in SaO_2_ from 100% to 75% in 30 seconds, 38% in 2 minutes and 32% in 3 minutes of apnea (Figure 2). Cardiac arrest occurred 6 minutes after disconnection.

### Case 2

A 43-year-old woman was admitted to the ICU with severe subarachnoid hemorrhage. Unresponsive coma, bilateral fixed dilated pupils and absence of all brainstem reflexes were found in two clinical examinations. Brain scintigraphy showed no radioisotope uptake into the brain parenchyma. The PaO_2_/FiO_2_ was 234, and there were no pulmonary infiltrates on chest radiography. Prior to the apnea test and after 10 minutes of preoxygenation at 100% FiO2, the following pretest blood gas analysis results were obtained: pH, 7.36; PaO_2_, 234.0mmHg; PaCO_2_, 46.8mmHg; HCO_3_, 18.7mmHg; and SaO_2_, 99.0%.

**Figure 3:**
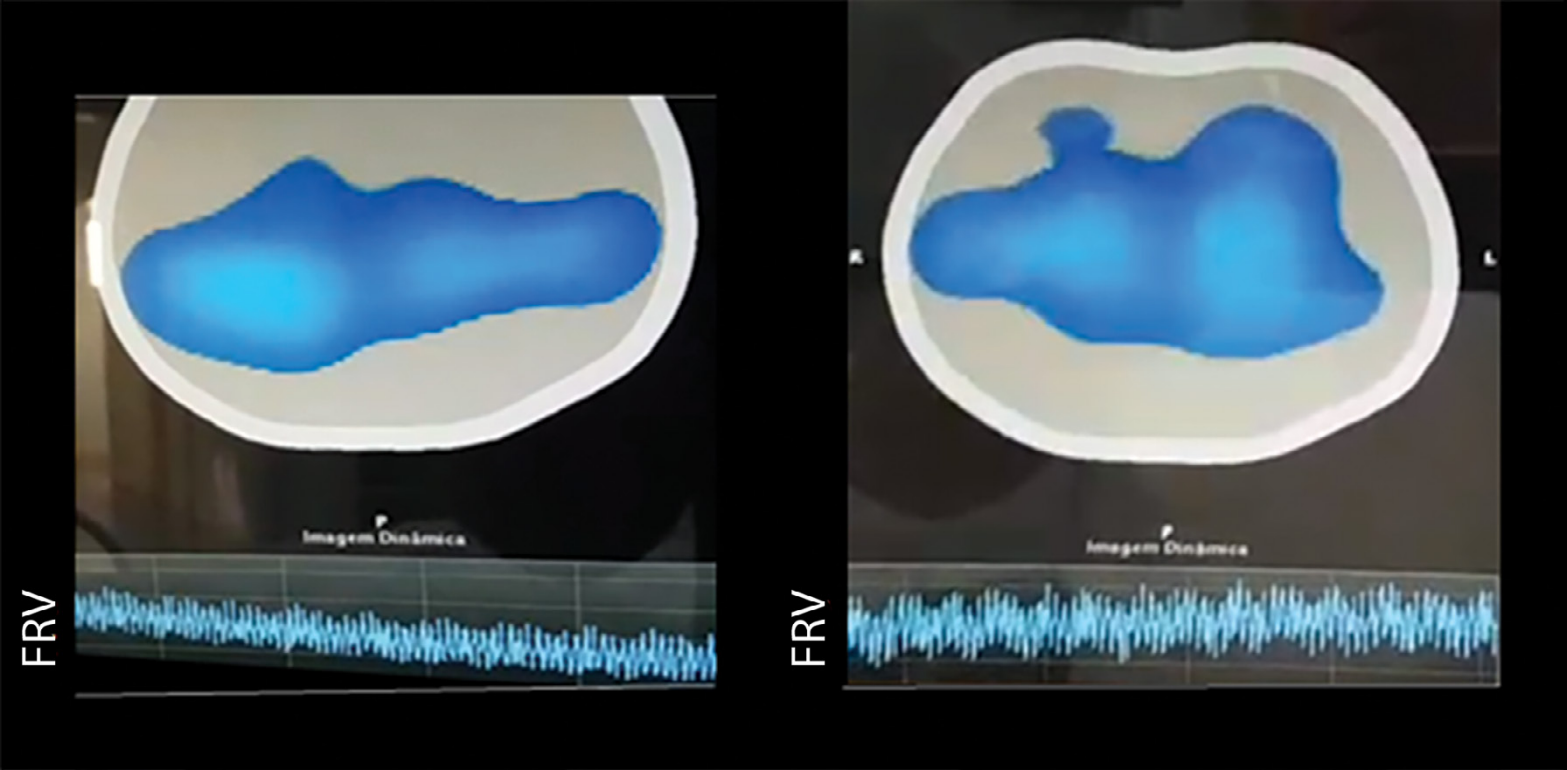
Chest electrical impedance tomography. (A) Gradual reduction in the functional residual volume after disconnection from the mechanical ventilator for the apnea test with oxygen infusion at 6 L/minute. (B) Maintenance of the functional residual volume provided by the apnea test without disconnection from the mechanical ventilator and maintenance of continuous airway pressure. FRV - functional residual volume.

The apnea test with MV disconnection was attempted. At the time of disconnection from the MV for the conventional test with oxygen infusion at 6 L/minute at the level of tracheal carina, the patient was monitored with chest electrical impedance tomography. Although the physiological prerequisites were met at the start of the apnea test (systolic blood pressure (SBP), 113 x 64mmHg; esophageal temperature, 35.1°C; and SaO_2_, 98%), there was a decrease in temperature to 34.9 °C at 5 minutes after disconnection from the MV. The procedure was interrupted to initiate body warming measures, including active warming of the MV gases. Electrical impedance tomography showed a progressive reduction in the functional residual volume (FRV) (Figure 3A).

After rewarming the patient, we chose to perform the apnea test without MV disconnection, setting the MV to spontaneous mode and maintaining 10cmH_2_O CPAP, 100% FiO_2_ and -2cmH_2_O trigger sensitivity. Electrical impedance tomography showed maintenance of the FRV (Figure 3B) and 100% SaO_2_ over 10 minutes, when the posttest blood gas analysis was performed, with the following results: pH, 7.01; PaO_2_, 232.0mmHg; PaCO_2_, 66.9mmHg; HCO_3_, 19.8mmHg; and SaO_2_, 99.0%. After the diagnosis of BD, the relatives authorized organ donation.

**Figure 4:**
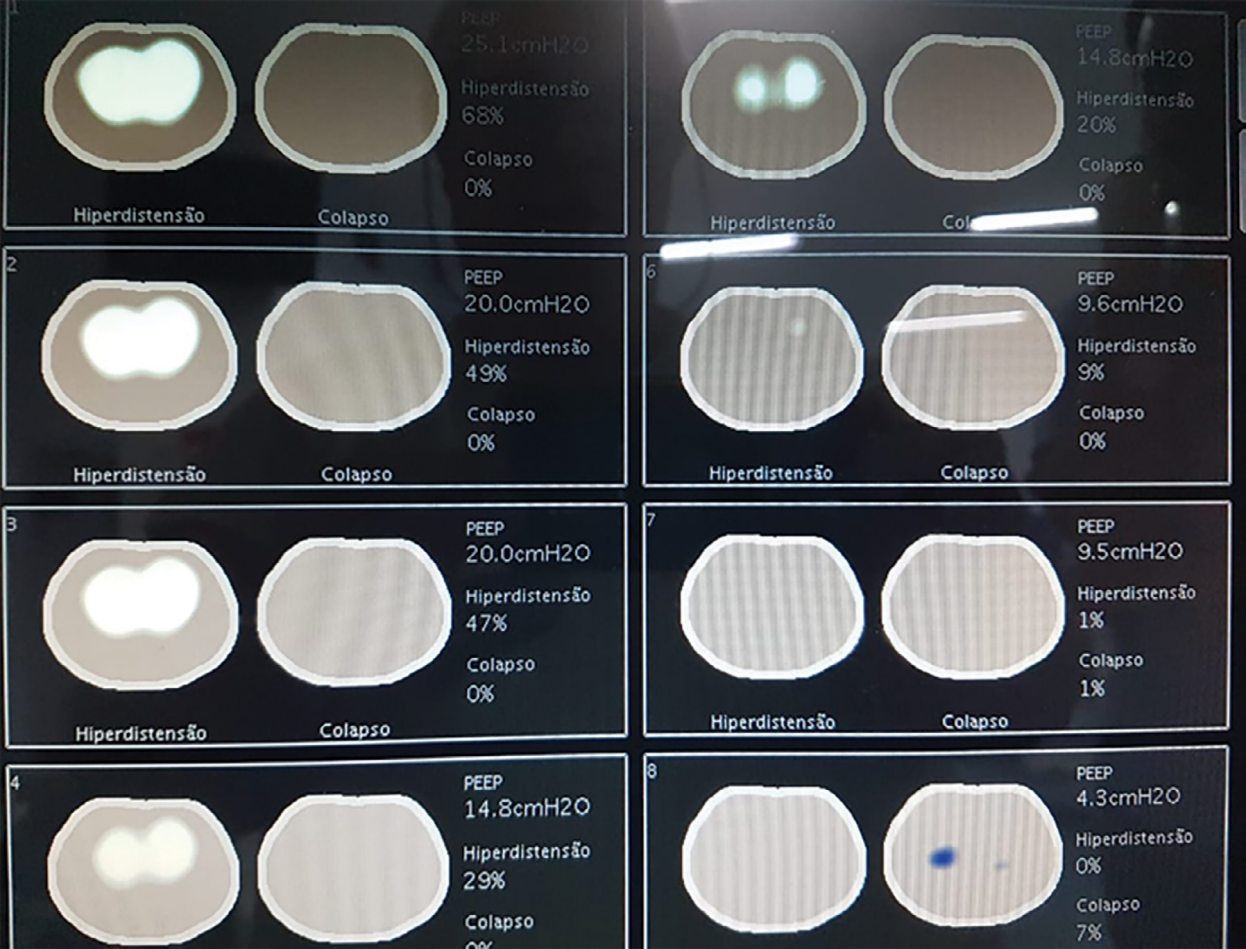
Chest electrical impedance tomography images indicating the percentage of hyperdistention and alveolar collapse, according to the level of positive endexpiratory pressure/continuous positive airway pressure applied. PEEP - positive end-expiratory pressure.

We used electrical impedance tomography to guide the titration of the expiratory pressure and found the best CPAP level. The lung hyperdistention and collapse map showed that the best balance of these variables occurred with 9.5cmH_2_O CPAP (1% hyperdistention and 1% collapse) (Figure 4).

Free and informed consent was provided by the relatives of both patients, and the present report was approved by a research ethics committee (CAAE: 10090919.1.000.5362). 

## DISCUSSION

The reported cases illustrate that the apnea test without disconnection from the MV in spontaneous mode and with application of CPAP allows maintenance of the FRV and of oxygenation throughout the test, even when there is prior hypoxemia.

The safety associated with the apnea test by CPAP was demonstrated in patients with normal lungs, in whom a decrease of 92mmHg in PaO_2_ was observed after the conventional test compared to a reduction of only 15mmHg in PaO_2_ after the test performed with the CPAP valve.^(5)^ Similarly, another study found a higher PaO_2_/FiO_2_ in apnea tests performed with a CPAP valve than with the conventional test (304 *versus* 250; p = 0.02).^(7)^

Some previous reports of apnea tests in individuals with ARDS reinforce the possibility of performing this procedure using different strategies in patients with severe hypoxemia.^(8-12)^ In a ventilated patient with ARDS, Hocker et al. performed an apnea test with the CPAP valve adjusted to the previously used 20cmH_2_O PEEP. The test was considered positive after finding apnea and 69mmHg PaCO_2_ at the end of 10 minutes. The SaO_2_ remained between 97% and 100%.^(8)^ In a patient with traumatic brain injury and ARDS due to pulmonary contusion, Ahlawat et al. gradually reduced the minute ventilation until reaching a PaCO_2_ of 99mmHg. Subsequently, the patient was observed for 60 seconds in CPAP mode. The absence of respiratory movements by hypercapnic stimulation of the respiratory center allowed completing the diagnosis of BD.^(10)^ Recently, a case was reported that showed the possibility of performing the apnea test in a prone position and with the CPAP valve coupled to a T-piece.^(11)^

In spite of previous hypoxemia observed in case 1, apnea testing with CPAP without disconnection from the MV allowed concluding BD with safety (Figure 3), which would not be possible with the conventional method, which presupposes disconnection from the ventilator. With the aid of electrical impedance tomography in the case 2, it was possible to visualize the effect of CPAP application on the preservation of the FRV, evidenced by the maintenance of chest impedance at the end of expiration (thoracic plethysmography with constant baseline) (Figure 3B). In contrast, during the conventional test, the thoracic plethysmography baseline was not maintained, which indicates a decrease in FRV (Figure 3A). Notably, electrical impedance tomography consists of measuring the impedance variation generated by the entrance and exit of air into and from the lungs, in addition to variations in blood content. During apnea, however, impedance variation occurs entirely by variations in blood content, making it impossible to assess ventilation distribution.^(16)^

Gas exchange is influenced by FiO_2_, by mean airway pressure and by the ventilation/perfusion ratio. Disconnection from the MV results in a reduction in airway pressure and FRV, which explains important declines in oxygenation during the conventional apnea test.^(17)^ Maintenance of the FRV provided by CPAP allows maintenance of the gas exchange surface. Thus, even with extensive injury to the alveolar-capillary membrane and low PaO_2_/FiO_2_ before the test, it is possible to maintain oxygen diffusion during the interruption of ventilation at levels similar to those observed in controlled ventilation.

Additionally to providing safety to patients with hypoxic respiratory failure, the apnea test with CPAP is being used as part of a lung preservation strategy for transplantation and includes a tidal volume of 6 to 8mL/kg and a PEEP of 8 to 10cmH_2_O, in addition to alveolar recruitment maneuvers.^(13-15)^ Although recruitment maneuvers^(8,11,13-15)^ are suggested by some studies, their potential complications must be considered, such as hypotension, decreased SaO_2_, arrhythmias and barotrauma. Also, maintenance of the FRV promoted by CPAP is likely sufficient to maintain oxygenation.^(10)^

## CONCLUSION

In hypoxemic neurocritical patients with suspected brain death, the conventional apnea test technique may result in severe hypoxemia. Performing the apnea test with the application of continuous airway pressure is a technique to be considered to prevent hypoxemia and maintain safety levels during the procedure.
